# Urolithiasis Prevalence and Associated Factors Among Adult Outpatients in Addis Ababa, Ethiopia: A Cross‐Sectional Study

**DOI:** 10.1155/bmri/4100205

**Published:** 2026-05-30

**Authors:** Mitiku Desalegn, Balkew Belay Girma, Yidnekachew Dedachew, Zelalem Negash, Tewoderos Shitemaw

**Affiliations:** ^1^ Department of Anesthesia, College of Medicine and Health Science, Wachemo University, Hossana, Ethiopia, wcu.edu.et; ^2^ Department of Public Health Emergency Management, Ethiopian Public Health Institute, Addis Ababa, Ethiopia, ephi.gov.et; ^3^ Department of Emergency Medicine and Critical Care, College of Medicine and Health Science, Wachemo University, Hossana, Ethiopia, wcu.edu.et; ^4^ School of Public Health, Yanet College, Addis Ababa, Ethiopia; ^5^ Department of Anesthesia, Menelik II College of Medical & Health Science, Addis Ababa, Ethiopia

**Keywords:** Addis Ababa, cross-sectional study, Ethiopia, kidney stones, prevalence, risk factors, urolithiasis

## Abstract

**Background:**

Urolithiasis represents a substantial global health concern with considerable morbidity. Limited epidemiological data exist regarding its prevalence and associated factors in Ethiopia, particularly in urban settings. This cross‐sectional study is aimed at determining the prevalence of urolithiasis and identifying associated risk factors among adult patients attending outpatient departments of selected public hospitals in Addis Ababa, Ethiopia.

**Methods:**

A hospital‐based cross‐sectional study was conducted from July 1 to August 15, 2024, among 220 adult patients systematically selected from three public hospitals in Addis Ababa. Data were collected using structured questionnaires and medical record reviews. Multivariate binary logistic regression was performed to identify independent predictors of urolithiasis, with statistical significance set at *p* < 0.05.

**Results:**

The prevalence of urolithiasis was 15.0% (33/220). In multivariate analysis, family history of kidney stones (AOR = 7.20, 95% CI: 2.78–18.65), sedentary lifestyle (AOR = 3.89, 95% CI: 1.43–10.63), regular alcohol consumption (AOR = 3.43, 95% CI: 1.36–8.68), and hyperlipidemia (AOR = 3.81, 95% CI: 1.09–13.35) were associated with increased odds of urolithiasis, with confidence intervals indicating moderate to strong positive associations. Conversely, adequate water intake (≥ 2 L/day) demonstrated a protective association (AOR = 0.35, 95% CI: 0.13–0.96).

**Conclusions:**

In this cross‐sectional study, urolithiasis affected a substantial proportion of adult outpatients in Addis Ababa. Associations were identified with genetic predisposition, modifiable lifestyle factors including physical inactivity, alcohol consumption, inadequate hydration, and metabolic abnormalities. These findings, while not demonstrating causality, underscore the need for targeted preventive strategies and further longitudinal research.

## 1. Introduction

Urolithiasis, characterized by the formation of calculi within the urinary tract, represents a major and growing global health challenge [1, 2]. The condition manifests across a spectrum of severity, ranging from asymptomatic microcalculi to acute episodes of renal colic and potentially life‐threatening complications including sepsis, obstructive uropathy, and renal failure [3]. Over recent decades, the global burden of urolithiasis has increased substantially, with prevalence estimates ranging from 1% to 20% across different populations: 7%–13% in North America, 5%–9% in Europe, and 1%–5% in Asia [4, 5].

While historically considered more prevalent in developed nations, urolithiasis is increasingly recognized as an emerging health concern in certain urban populations undergoing rapid lifestyle transitions, attributed to urbanization, dietary transitions, and changing lifestyle patterns [6, 7]. The condition results from a complex interplay of genetic predisposition, environmental exposures, dietary habits, fluid intake, and metabolic abnormalities. Understanding local epidemiological patterns and risk factors is essential for developing targeted preventive strategies and optimizing patient management [8, 9].

In Ethiopia, evidence regarding urolithiasis epidemiology remains limited despite anecdotal reports of increasing case presentations at healthcare facilities [10, 11]. Previous studies have primarily focused on clinical presentations and surgical outcomes at tertiary centers, with limited investigation of community‐level prevalence and associated factors [12, 13].

Addis Ababa, as the capital and largest urban center, presents a unique context where lifestyle transitions and healthcare access patterns may influence disease occurrence. However, specific data on the burden and determinants of urolithiasis among patients seeking care in public hospital outpatient departments remain scarce. Therefore, the objectives of this study were (1) to determine the prevalence of urolithiasis among adult patients attending outpatient departments of selected public hospitals in Addis Ababa, Ethiopia, and (2) to identify sociodemographic, behavioral, and clinical factors associated with urolithiasis in this population. The findings are intended to inform evidence‐based preventive interventions and guide healthcare policy development for urolithiasis management in similar urban African settings. The sample in this study was predominantly from relatively affluent socioeconomic backgrounds and demonstrated an association with hyperlipidemia; therefore, the findings are not representative of the general Ethiopian population or of typical low‐income settings.

## 2. Methods

### 2.1. Study Design and Setting

A hospital‐based cross‐sectional study was conducted from July 1 to August 15, 2024, in Addis Ababa, the capital city of Ethiopia. Addis Ababa covers approximately 527 km^2^ and comprises 11 subcities, with an estimated population of 4.59 million in 2019. The city has 12 public hospitals, of which seven are administered by the Addis Ababa Health Bureau and five by the Federal Ministry of Health.

Three hospitals (Menelik II Referral Hospital, Ras Desta Damtew Hospital, and Zewditu Memorial Hospital) were purposively selected based on their geographic distribution, patient volume, availability of urology services, and administrative willingness to participate. These facilities serve diverse patient populations from various subcities and surrounding regions.

### 2.2. Participants

#### 2.2.1. Eligibility Criteria

The source population comprised all adult patients aged 18 years and above seeking healthcare services at the outpatient departments of the selected hospitals during the study period.

#### 2.2.2. Inclusion Criteria

The inclusion criteria are adult patients aged ≥ 18 years who attended the outpatient departments during the study period and provided informed consent.

#### 2.2.3. Exclusion Criteria

The exclusion criteria are patients aged < 18 years, individuals unable to provide informed consent due to cognitive impairment or language barriers (Amharic and English were the languages used), and critically ill patients requiring emergency care.

### 2.3. Variables

#### 2.3.1. Outcome Variable

Urolithiasis was defined as physician‐diagnosed urinary tract stones documented in the patient’s medical chart, confirmed by imaging (ultrasonography, radiography, or computed tomography) when available. This definition follows standard clinical practice.

#### 2.3.2. Independent Variables

Data were collected on the following:

• Sociodemographic characteristics: age, sex, marital status, educational level, occupation, and monthly income
• Behavioral factors: cigarette smoking status, alcohol consumption, physical activity level, and daily water intake
• Clinical characteristics: body mass index (BMI), family history of urolithiasis, personal history of recurrent stones, comorbidities (hypertension, diabetes mellitus, hyperlipidemia, gout, chronic kidney disease, urinary tract infections, and malignancies), and presenting symptoms

### 2.4. Data Sources and Measurement

Data were collected using a structured questionnaire adapted from standard national patient information systems and validated through literature review. The questionnaire was developed in English, translated into Amharic, and back‐translated to ensure consistency. Four trained data collectors (health professionals, including two nurses and two health officers) administered questionnaires through face‐to‐face interviews in private rooms within the outpatient departments and reviewed medical records to confirm diagnoses and clinical data. Each interview lasted approximately 15–20 min. Physical activity was categorized according to WHO guidelines [14]. BMI was calculated from measured weight and height using calibrated scales and stadiometers.

### 2.5. Bias

To reduce information bias, data collectors received 1 day of training on standardized interview techniques. To reduce social desirability bias, participants were assured of confidentiality and that responses would not affect their medical care. To reduce selection bias, systematic random sampling was employed with a random start. Recall bias for behavioral factors (water intake, physical activity, and alcohol consumption) is a recognized limitation of self‐reported data. Diagnostic confirmation bias was minimized by using medical record documentation rather than self‐report for the outcome.

### 2.6. Study Size and Sampling Procedure

Sample size was calculated a priori using the single population proportion formula, assuming a 15% prevalence of urolithiasis based on previous research [7], 95% confidence level, and 5% margin of error:

*n* = (*Z* *α*/2)^2^ × *p*(1 − *p*)/*d* ^2^ = (1.96)^2^ × 0.15(0.85)/(0.05)^2^ = 196.

Accounting for a 10% nonresponse rate, the final sample size was 220 participants.

**Figure 1:**
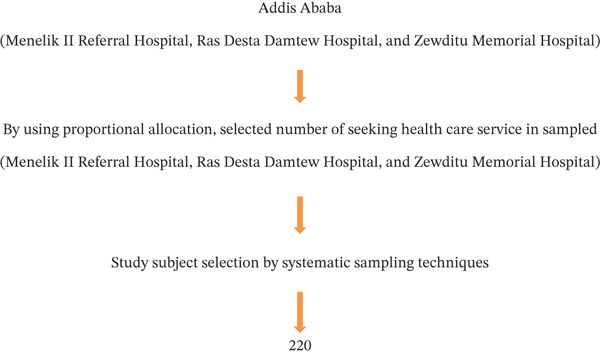
Schematic presentation of sampling procedures.

A two‐stage sampling approach was employed. First, three hospitals were randomly selected from the 12 public hospitals in Addis Ababa using simple random sampling. Second, study participants were selected using systematic random sampling from each hospital’s outpatient department, with proportional allocation based on the average daily patient flow. The sampling interval was determined by dividing the total estimated weekly OPD attendance by the required sample size for each facility. The first participant was randomly selected, and subsequent participants were chosen at regular intervals (Figure 1).

### 2.7. Quantitative Variables

Age was analyzed both as a continuous variable (mean, SD) and categorized for clinical interpretability. BMI was similarly analyzed continuously and categorically. Water intake was dichotomized at 2 L/day based on standard clinical recommendations. Physical activity is dichotomized as sedentary versus nonsedentary per WHO criteria [14].

### 2.8. Statistical Methods

Data were entered into EpiData Version 3.1 and exported to SPSS Version 26 for analysis. Descriptive statistics, including frequencies, proportions, means, and standard deviations, were calculated to summarize participant characteristics. Bivariate logistic regression analysis was performed to examine associations between each independent variable and urolithiasis. Variables with *p* < 0.25 in bivariate analysis were candidates for the multivariate logistic regression model to control for potential confounding. This threshold was chosen to avoid excluding potentially important variables. Adjusted odds ratios (AORs) with 95% confidence intervals (CIs) were calculated, and statistical significance was declared at *p* < 0.05. In line with recommendations against dichotomization of results, we emphasize interpretation of the point estimates and the bounds of the CIs rather than relying solely on *p* values. To address multiplicity arising from testing multiple associations, we focus on the magnitude and precision of each effect estimate, recognizing that *p* values are descriptive and should not be interpreted as strict significance tests. Model fitness was assessed using the Hosmer–Lemeshow goodness‐of‐fit test (*p* = 0.67, indicating good fit). Multicollinearity was checked using variance inflation factors (all VIFs < 2.0). Missing data were minimal (< 2% for any variable) and handled by listwise deletion. As this is a cross‐sectional study, all reported associations are descriptive and should not be interpreted as causal; the analysis is aimed at describing the magnitude and direction of observed associations rather than at establishing causality.

## 3. Results

### 3.1. Sociodemographic Characteristics

A total of 220 eligible participants were enrolled, yielding a 100% response rate. The study population comprised 115/220 (52.3%) males and 105/220 (47.7%) females, with a male‐to‐female ratio of 1.1:1. The mean age was 48.1 years (SD = 15.5 years; range: 19–88 years). The largest age group was 45–54 years (52/220, 23.6%), followed by 35–44 years (48/220, 21.8%) and 55–64 years (39/220, 17.7%).

The majority of participants were married (166/220, 75.5%), while 34/220 (15.5%) were single, 15/220 (6.8%) divorced, and 5/220 (2.3%) widowed. Regarding educational attainment, 91/220 (41.4%) had completed university education or higher, 86/220 (39.1%) had secondary education, and 43/220 (19.5%) had primary education or below. Occupations varied, with government employees (58/220, 26.4%) and private sector workers (54/220, 24.5%) being most common, followed by self‐employed individuals (33/220, 15.0%), housewives (28/220, 12.7%), unemployed persons (23/220, 10.5%), and students (8/220, 3.6%). Monthly income exceeded 5000 Ethiopian Birr for 134/220 (60.9%) of participants, while 47/220 (21.4%) earned 2500–5000 Birr, and 39/220 (17.7%) earned less than 2500 Birr (Table 1).

**Table 1:** Cross‐tabulation of sociodemographic characteristics of study participants (*N* = 220).

Variables	Category	Presence of urolithiasis (*N* = 220)		Total	Percent (%)
		No (187)	Yes (33)		
Gender	Female	89	16	105	47.7%
	Male	98	17	115	52.3%

Age category	65 and above	26	10	36	16.4%
	Between 18 and 24	10	0	10	4.5%
	Between 25 and 34	31	4	35	15.9%
	Between 35 and 44	45	3	48	21.8%
	Between 45 and 54	44	8	52	23.6%
	Between 55 and 64	31	8	39	17.7%

Occupation	Government	47	11	58	26.4%
	House wife	24	4	28	12.7%
	Nongovernmental	14	2	16	7.3%
	Private	48	6	54	24.5%
	Self‐employed	28	5	33	15.0%
	Student	8	0	8	3.6%
	Unemployed	18	5	23	10.5%

Educational status	Primary or below	38	5	43	19.5%
	Secondary	71	15	86	39.1%
	University or above	78	13	91	41.4%

Monthly income	> 5000	113	21	134	60.9%
	2500–5000	41	6	47	21.4%
	Less than 2500	33	6	39	17.7%

Marital status	Divorced	11	4	15	6.8%
	Married	143	23	166	75.5%
	Single	30	4	34	15.5%
	Windowed	3	2	5	2.3%
	Yes	31	13	44	20.0%

Physical activity	Nonsedentary life	157	21	178	80.9%
	Sedentary lifestyle	30	12	42	19.1%

Abbreviations: BMI = body mass index, OPD = outpatient department.

### 3.2. Behavioral and Lifestyle Characteristics

Only 44/220 (20.0%) of participants reported consuming the recommended daily water intake of ≥ 2 L, while 176/220 (80.0%) consumed less than this amount. Regular alcohol consumption was reported by 31/220 (14.1%) of participants, and cigarette smoking by 18/220 (8.2%). Regarding physical activity, 178/220 (80.9%) led an active (nonsedentary) lifestyle, while 42/220 (19.1%) had a sedentary lifestyle (Table 1).

### 3.3. Clinical Characteristics

BMI classification revealed that 115/220 (52.3%) of participants had normal weight, 86/220 (39.1%) were preobese (overweight), 15/220 (6.8%) were obese, and 4/220 (1.8%) were underweight. A family history of kidney stones or urolithiasis was reported by 34/220 (15.5%) of participants, and 11/220 (5.0%) had a history of recurrent kidney stones.

Comorbidities were present in 86/220 (39.1%) of participants. The most common comorbid conditions were hypertension (37/220, 16.8%), diabetes mellitus (33/220, 15.0%), malignancies (19/220, 8.6%), hyperlipidemia (16/220, 7.3%), gout (7/220, 3.2%), and chronic kidney disease (4/220, 1.8%). Additionally, 91/220 (41.4%) of participants reported a history of urinary tract infections.

Among participants with urolithiasis (*n* = 33), presenting symptoms included back pain (4/33, 12.1%), frequent urination (2/33, 6.1%), dysuria (2/33, 6.1%), hematuria (1/33, 3.0%), nausea (1/33, 3.0%), and vomiting (1/33, 3.0%). Hydronephrosis was documented in 2/220 (0.9%) of all participants (2/33, 6.1% of stone formers) (Table 2).

**Table 2:** Cross‐tabulation of health‐related characteristics of participants (*N* = 220).

Variables	Category	Presence of urolithiasis (*N* = 220)		Total	%
		No (187)	Yes (33)		
BMI category	Obesity	13	2	15	6.8%
	Preobesity	69	17	86	39.1%
	Underweight	2	2	4	1.8%
	Normal weight	103	12	115	52.3%
Family history of kidney stones or urolithiasis?	No	168	18	186	84.5%
	Yes	19	15	34	15.5%
Recurrent kidney stones	No	187	22	209	95.0%
	Yes	0	11	11	5.0%
Comorbidity	No	119	15	134	60.9%
	Yes	68	18	86	39.1%
Have you ever diagnosed for urinary tract infection	No	112	17	129	58.6%
	Yes	75	16	91	41.4%
Any malignancies	No	169	32	201	91.4%
	Yes	18	1	19	8.6%
Diabetes mellitus	No	163	24	187	85.0%
	Yes	24	9	33	15.0%
Presence of hyperlipidemia	No	178	26	204	92.7%
	Yes	9	7	16	7.3%
Presence of gout	No	181	32	213	96.8%
	Yes	6	1	7	3.2%
Presence of hypertension	No	161	22	183	83.2%
	Yes	26	11	37	16.8%
Presence of chronic kidney disease	No	184	32	216	98.2%
	Yes	3	1	4	1.8%
Hydronephrosis	No	187	31	218	99.1%
	Yes	0	2	2	0.9%

Abbreviation: BMI = body mass index.

### 3.4. Prevalence of Urolithiasis

The overall prevalence of urolithiasis among study participants was 15.0% (33/220). Among those with urolithiasis, 17/33 (51.5%) were male, and 16/33 (48.5%) were female.

### 3.5. Factors Associated With Urolithiasis

#### 3.5.1. Bivariate Analysis

In bivariate logistic regression analysis, several factors showed associations with urolithiasis at *p* < 0.25 and were subsequently included in the multivariate model: age category, underweight BMI, family history of kidney stones, physical inactivity, hyperlipidemia, alcohol consumption, water intake, and history of urinary tract infection.

#### 3.5.2. Multivariate Analysis

The multivariate binary logistic regression analysis identified five factors associated with urolithiasis (Table 3; interpretation focuses on point estimates and CI bounds).

**Table 3:** Multivariate regression conducted after bivariate analysis done.

		Urolithiasis		*p*value	AOR (CI)	95% CI	
		No (187)	Yes (33)				
BMI category recoded underweight	Not Underweight	185	31	1	1		
	Underweight	2	2	0.052	7.974	0.986	64.471
Family history of kidney stones or urolithiasis?	No	168	18	1	1		
	Yes	19	15	0.000	7.203	2.782	18.646
Physical activity	Nonsedentary lifestyle	157	21	1	1		
	Sedentary lifestyle	30	12	0.008	3.893	1.426	10.629
Hyperlipidemia	No	178	26	1	1		
	Yes	9	7	0.036	3.813	1.089	13.352
Alcohol consumption	No	159	20	1	1		
	Yes	28	13	0.009	3.433	1.357	8.683
How many glasses of water do you typically drink per day	2 L and above	33	11	1	1		
	Less than 2 L	154	22	0.040	0.351	0.129	0.955

*Note:* Reference category for each variable is indicated by AOR = 1.

Abbreviations: AOR = adjusted odds ratio, CI = confidence interval.

Family history of kidney stones or urolithiasis showed the strongest positive association, with individuals reporting a positive family history having an AOR of 7.20 (95% CI: 2.78–18.65). The CI ranges from a 2.8‐fold to an 18.7‐fold increase, indicating a consistently positive association across plausible values.

Sedentary lifestyle was associated with increased odds of urolithiasis. Participants with sedentary behavior had an AOR of 3.89 (95% CI: 1.43–10.63) compared to physically active individuals. The lower bound of the CI (1.43) suggests that the association is unlikely to be null or protective.

Regular alcohol consumption showed an AOR of 3.43 (95% CI: 1.36–8.68), with the CI indicating a positive association ranging from approximately 1.4‐fold to 8.7‐fold increased odds.

Hyperlipidemia demonstrated an AOR of 3.81 (95% CI: 1.09–13.35). Although the CI is relatively wide—reflecting some imprecision due to the small number of hyperlipidemic cases—the lower bound remains above 1.0, suggesting a positive association.

Conversely, adequate water intake (≥ 2 L/day) showed a protective association. Participants consuming less than the recommended daily water intake had an AOR of 0.35 (95% CI: 0.13–0.96) compared to those with adequate intake, indicating that lower water intake is associated with reduced odds of urolithiasis. The CI (0.13–0.96) excludes 1.0, supporting the direction of this association.

While being underweight showed an AOR of 7.97 (95% CI: 0.99–64.47), the CI is very wide and includes values close to 1.0 (lower bound 0.99), indicating considerable imprecision; thus, this association is less certain and should be interpreted cautiously (Table 3).

## 4. Discussion

This study investigated the prevalence and associated factors of urolithiasis among adult patients attending outpatient departments of public hospitals in Addis Ababa, Ethiopia. The observed prevalence of 15.0% indicates that urolithiasis represents a substantial health concern in this urban Ethiopian population. However, given the cross‐sectional design of this study, the identified associations should not be interpreted as causal; they reflect statistical relationships that require confirmation through longitudinal or experimental designs. This finding aligns with global prevalence estimates ranging from 1% to 20% [4, 5] and is consistent with the 15% prevalence reported in previous Ethiopian research [7]. The prevalence falls within the intermediate range compared to North American (7%–13%) and European (5%–9%) estimates [4], suggesting that urbanization and lifestyle transitions in Addis Ababa may be contributing to disease burden comparable to some developed settings.

### 4.1. Genetic Predisposition

The strongest predictor of urolithiasis in this study was family history, with affected individuals demonstrating more than sevenfold increased odds. This finding robustly supports the substantial genetic component of urolithiasis pathogenesis, consistent with previous research documenting hereditary patterns in stone formation [8, 15]. Genetic predisposition may operate through various mechanisms, including inherited metabolic disorders such as hypercalciuria, hyperoxaluria, cystinuria, and distal renal tubular acidosis [16]. The magnitude of this association underscores the importance of obtaining detailed family histories during routine clinical assessments and considering targeted preventive strategies for high‐risk individuals. Genetic counseling and early metabolic evaluation may be warranted for those with affected first‐degree relatives.

### 4.2. Lifestyle Factors

The significant association between a sedentary lifestyle and urolithiasis (nearly fourfold increased odds) adds to growing evidence linking physical inactivity with kidney stone risk [17, 18]. Physical activity may protect against stone formation through multiple mechanisms: promoting regular urine flow, reducing urinary stasis, maintaining healthy body weight, improving insulin sensitivity, and modifying urinary composition [19]. Conversely, prolonged sedentary behavior, increasingly prevalent in urban settings, may contribute to urinary supersaturation and crystal retention. These findings support the integration of physical activity promotion into urolithiasis prevention strategies, aligning with WHO recommendations for regular physical activity to maintain health [14].

Alcohol consumption emerged as a notable risk factor, with regular drinkers showing more than threefold increased odds of urolithiasis. While the relationship between alcohol and stone formation is complex and somewhat understudied, plausible mechanisms include alcohol‐induced dehydration, altered urinary pH, increased uric acid production, and modifications in calcium and oxalate excretion [18]. Binge drinking patterns, common in some social contexts, may be particularly problematic due to acute dehydration effects [20]. These findings suggest that public health messages should address alcohol moderation as a component of kidney stone prevention, particularly in populations where alcohol consumption is prevalent [21].

The protective effect of adequate water intake demonstrated in this study reinforces the well‐established role of hydration in preventing urolithiasis [22, 23]. Participants consuming less than the recommended 2 L daily had significantly reduced odds of stones, consistent with extensive literature showing that high fluid intake dilutes urinary stone‐forming constituents, reduces supersaturation, and promotes crystal excretion [24]. The finding that only 20% of participants met recommended water intake targets highlights a critical opportunity for public health intervention. In Addis Ababa’s climate, where ambient temperatures can be high and dehydration risk is increased, promoting adequate hydration should be a cornerstone of preventive strategies [20].

### 4.3. Metabolic Factors

The observed association between hyperlipidemia and urolithiasis (nearly fourfold increased odds) contributes to growing recognition of kidney stones as a metabolic disorder linked to the metabolic syndrome [25, 26]. Hyperlipidemia may influence stone formation through several pathways: alterations in urinary lipid composition affecting crystal nucleation and aggregation, associations with obesity and insulin resistance affecting urinary pH and citrate excretion, and shared dietary risk factors [27]. This finding underscores the importance of comprehensive metabolic assessment in stone formers and suggests that lipid‐lowering interventions, including dietary modifications and pharmacotherapy when indicated, may have dual benefits for cardiovascular and renal stone prevention.

While hypertension and diabetes mellitus showed trends toward increased stone risk, these associations did not reach statistical significance, possibly due to sample size limitations. However, the clustering of metabolic abnormalities observed suggests that urolithiasis should be considered within the broader context of cardiometabolic health, and preventive strategies should address multiple risk factors simultaneously [28–30].

### 4.4. Weight Extremes and Urolithiasis

The near‐borderline association between underweight status and urolithiasis (*p* = 0.052) is intriguing and contrasts with the more commonly reported obesity‐stone association [31]. This finding may reflect specific metabolic disturbances in underweight individuals, such as altered urinary composition, reduced protective factors, or underlying malabsorptive conditions that promote stone formation [32]. Alternatively, it might represent reverse causality if chronic stone disease leads to dietary restrictions and weight loss. This observation warrants further investigation in larger studies and suggests that weight management interventions for stone prevention should consider both extremes of the weight spectrum.

### 4.5. Comparison With Regional Studies

The 15% prevalence observed in this study is higher than the 10.5% prevalence reported in a Kenyan study [33] but lower than the 20% reported in some Middle Eastern populations [34]. These variations likely reflect differences in study populations, diagnostic methods, dietary patterns, climate, and genetic backgrounds. The significant associations with family history, lifestyle factors, and metabolic abnormalities are consistent with findings from other African [35] and global studies [36], suggesting that similar risk factor profiles operate across diverse settings.

### 4.6. Public Health Implications

The findings of this study have several important public health implications for Addis Ababa and similar urban African settings:

1. Screening and risk stratification: Healthcare providers should routinely assess family history of kidney stones and consider targeted metabolic evaluation for high‐risk individuals.
2. Health education: Public health campaigns should emphasize the importance of adequate hydration (≥ 2 L daily), regular physical activity, and alcohol moderation for kidney stone prevention.
3. Metabolic health promotion: Given the association with hyperlipidemia and clustering of metabolic risk factors, comprehensive lifestyle interventions addressing diet, physical activity, and weight management may reduce stone burden while improving overall cardiometabolic health.
4. Healthcare system preparedness: The substantial prevalence of urolithiasis suggests a need for adequate diagnostic imaging capabilities, urology services, and trained personnel in public hospitals.
5. Policy development: These findings can inform evidence‐based guidelines for urolithiasis prevention and management in Ethiopian healthcare settings.

### 4.7. Strengths and Limitations

This study has several strengths, including its focus on an understudied population, use of validated data collection instruments, robust sampling methodology, and multivariate analysis controlling for potential confounders. The inclusion of multiple hospitals enhances generalizability within Addis Ababa.

However, several limitations should be acknowledged. First, the cross‐sectional design precludes establishment of causal relationships between identified factors and urolithiasis. Second, reliance on self‐reported behavioral data (water intake, physical activity, and alcohol consumption) may be subject to recall and social desirability biases. Third, the study was conducted in public hospital outpatient settings, and findings may not generalize to community populations or private healthcare facilities. Fourth, stone composition analysis was not available, preventing investigation of risk factor variations by stone type. Fifth, the small sample size, while adequate for primary objectives, may have limited power to detect associations for less prevalent exposures and limits the generalizability of the findings. Sixth, the exclusion of patients unable to provide consent due to language barriers may have introduced selection bias. Additionally, the study did not capture data on stone morphology, specific anatomical location, or whether active treatment was required. The absence of these variables limits the clinical depth of our findings and prevents risk stratification based on stone characteristics.

Despite these limitations, this study provides valuable baseline data on urolithiasis epidemiology in Addis Ababa and identifies modifiable risk factors that can be targeted through public health interventions.

## 5. Conclusions

This study demonstrates that urolithiasis affects a substantial proportion (15.0%, 33/220) of adult outpatients in Addis Ababa public hospitals. Genetic predisposition, reflected in family history (34/220, 15.5% of all participants; 15/33, 45.5% of stone formers), represents the strongest risk factor, underscoring the importance of hereditary influences in stone pathogenesis. Crucially, several modifiable factors considerably influence disease occurrence: physical inactivity (42/220, 19.1% of all participants; 12/33, 36.4% of stone formers), alcohol consumption (31/220, 14.1% of all participants; 13/33, 39.4% of stone formers), inadequate hydration (176/220, 80.0% of all participants), and hyperlipidemia (16/220, 7.3% of all participants; 7/33, 21.2% of stone formers) each independently increased stone risk, while adequate water intake provided meaningful protection.

These findings highlight the multifaceted nature of urolithiasis risk and support comprehensive prevention strategies addressing both nonmodifiable genetic factors and modifiable lifestyle and metabolic parameters. In clinical practice, routine assessment of family history and metabolic risk factors, coupled with targeted counseling on hydration, physical activity, alcohol moderation, and lipid management, may substantially reduce individual stone risk. At the population level, public health interventions promoting healthy lifestyles and adequate hydration could significantly decrease the community burden of urolithiasis.

Future research should focus on longitudinal studies to establish causal relationships, investigate stone composition patterns in Ethiopian populations, evaluate the effectiveness of preventive interventions, and explore genetic markers of susceptibility. Additionally, studies in rural and diverse geographic settings within Ethiopia would provide a more comprehensive understanding of national urolithiasis epidemiology. Addressing these knowledge gaps will facilitate development of evidence‐based, culturally appropriate strategies for urolithiasis prevention and management in Ethiopia and similar resource‐limited settings.

## Author Contributions

B.B.G. and M.D. came up with the study, created the method, did the analysis, and wrote the manuscript. T.S., Y.D., and Z.N. helped with supervision, statistical analysis, interpretation, and reviewing the manuscript.

## Funding

This research did not receive a specific grant from any funding agencies in the public, commercial, or nonprofit sectors.

## Disclosure

All authors read and approved the final manuscript.

## Ethics Statement

Ethical approval was obtained from the Institutional Review Board of Yanet College under protocol number 0222/2024. Permission was granted by Addis Ababa City Administration Health Bureau and the participating hospitals. Additional permissions were secured from the administrative bodies of each participating hospital. Written informed consent was obtained from all participants after explaining the study purpose, procedures, potential risks, and benefits. Confidentiality was maintained throughout by using unique identifiers instead of personal names and securing data in password‐protected files. Participants were informed of their right to withdraw at any time without affecting their medical care. The study was carried out in line with the Declaration of Helsinki (https://www.wma.net/policies-post/wma-declaration-of-helsinki/).

## Consent

The authors declare that written informed consent for publication of relevant data and laboratory result has been secured from all participants. Participants’ identities have been kept confidential.

## Conflicts of Interest

The authors declare no conflicts of interest.

## Data Availability

The datasets used and/or analyzed during the current study are available from the corresponding author on reasonable request.
